# Amplicon-Based Sequencing of Soil Fungi from Wood Preservative Test Sites

**DOI:** 10.3389/fmicb.2017.01997

**Published:** 2017-10-18

**Authors:** Grant T. Kirker, Amy B. Bishell, Michelle A. Jusino, Jonathan M. Palmer, William J. Hickey, Daniel L. Lindner

**Affiliations:** ^1^FPL, United States Department of Agriculture-Forest Service (USDA-FS), Durability and Wood Protection, Madison, WI, United States; ^2^NRS, United States Department of Agriculture-Forest Service (USDA-FS), Center for Forest Mycology Research, Madison, WI, United States; ^3^Department of Soil Science, University of Wisconsin-Madison, Madison, WI, United States

**Keywords:** amplicon sequencing, environmental impacts, DNA, soil fungal communities, wood decay fungi, wood preservatives

## Abstract

Soil samples were collected from field sites in two AWPA (American Wood Protection Association) wood decay hazard zones in North America. Two field plots at each site were exposed to differing preservative chemistries via in-ground installations of treated wood stakes for approximately 50 years. The purpose of this study is to characterize soil fungal species and to determine if long term exposure to various wood preservatives impacts soil fungal community composition. Soil fungal communities were compared using amplicon-based DNA sequencing of the internal transcribed spacer 1 (ITS1) region of the rDNA array. Data show that soil fungal community composition differs significantly between the two sites and that long-term exposure to different preservative chemistries is correlated with different species composition of soil fungi. However, chemical analyses using ICP-OES found levels of select residual preservative actives (copper, chromium and arsenic) to be similar to naturally occurring levels in unexposed areas. A list of indicator species was compiled for each treatment-site combination; functional guild analyses indicate that long-term exposure to wood preservatives may have both detrimental and stimulatory effects on soil fungal species composition. Fungi with demonstrated capacity to degrade industrial pollutants were found to be highly correlated with areas that experienced long-term exposure to preservative testing.

## Introduction

Wood preservation as an industry has a long-standing history of using persistent chemicals to prevent colonization of treated wood by decay fungi and insects. Chemically treated wood in contact with soil has the potential to expose the surrounding soil and its inhabitants to these chemicals through chemical migration. Previous studies have shown that wood preservatives can migrate from treated wood, but environmental exposure is typically very low and varies based on preservative chemistry, environmental exposure and soil composition (Lebow et al., 2004). The effects of prolonged wood preservation chemical exposure on soil fungal communities is not fully understood (Bhattacharya et al., 2002). Soil fungal communities are key drivers of soil geochemical processes and assist in cycling of carbon, nitrogen, and other nutrients (Tedersoo et al., 2014). Classical biodiversity studies based on fungal fruiting body surveys are limited, as uncultivable fungi can constitute a major proportion of the total micro-biota of a given location (Gams, 2007). Thus, DNA-based identification of fungal communities has been a valuable tool to assess fungal diversity (Schoch et al., 2012). Coupling DNA-based identification methods and next-generation sequencing has proven to be a powerful tool to identify fungi within environmental samples and subsequently compare these samples under different experimental conditions (Daniel, 2005).

Previous studies have found that soil fungal diversity is in fact very homogeneous in deeper soil horizons, consisting mostly of ectomycorrhizal and other root associated fungi (Dickie et al., 2002). However, the litter layer, found in the first few inches of soil strata, holds the key components of soil metabolic capabilities (Voriskova and Baldrian, 2013). The rate at which biomass is turned over through biochemical breakdown and subsequent nutrient cycling has been found to vary considerably with respect to soil fungal diversity and structure (Abrego and Salcedo, 2013). Therefore, the effects of wood preservative migration into surrounding soil communities has the potential to impact fungal species with varying nutrient cycling strategies.

Environmental impacts of wood preservatives are a major concern of the industry; end users have been a driving force behind the ever changing spectrum of preservatives used to protect wood in service (Schultz et al., 2007). Early preservative systems relied on broad spectrum toxins that restricted the growth of deteriorative agents with little or no consideration of the impacts of these compounds on the surrounding environment (Connell, 1991). More recent preservative systems have been more targeted in an attempt to minimize broad environmental impacts (Schultz and Nicholas, 2002). First generation preservation systems consisted of mostly creosote, some highly toxic metals such as mercury, cadmium and arsenic and relied on broad spectrum control of microbes, including bacteria, which have been shown to contribute to wood permeability and preservative breakdown (Schultz et al., 2007). Chemical migration of wood preservatives in use has also been a driver of change based on consumer concerns, which has led to some voluntary restrictions on their use by the wood preservation industry (Hingston et al., 2001). The potential negative health effects of these toxic metal compounds greatly outweighed their efficacy as preservatives; mercury and cadmium are both highly water soluble and arsenic in high dosages is toxic to most life forms (Hughes, 2002). Newer preservative formulations have much reduced risks associated with long-term exposure and consist of copper combined with an organic co-biocide. These have been shown to have good activity against most wood decay fungi (Freeman and Mcintyre, 2008). A persistent problem that can severely impact the service life of copper treated lumber is the presence of copper tolerant fungi, which often arise either due to genetic predisposition of fungi to detoxify copper or as a result of long-term exposure to a copper rich environment (Clausen and Green, 2003). Several known wood decay fungi are classified as copper tolerant and have been studied in order to understand mechanisms of copper tolerance (Green and Clausen, 2005).

While there is substantial literature depicting changes in soil fungal biota due to metal contaminates (Kandeler et al., 1996; Giller et al., 1998; Bhattacharya et al., 2002; Rajapaksha et al., 2004; Oliveira and Pampulha, 2006), little is known about changes in soil fungal biodiversity as a specific result of prolonged wood preservative exposure and few studies have employed amplicon based sequencing to compare soil fungal communities under after exposure. Prior studies have shown that preservatives can migrate from wood in ground contact (Lebow et al., 2004), but these concentrations are much less than the amounts present in the wood and are typically only within 5–10 cm of the treated material. Kirker et al. (2012) found differences in the fungal diversity and colonization patterns of ammoniacal copper quaternary type C (ACQ-C) treated pine in field studies, but those differences became less pronounced after longer exposure (12 months). The primary objective of this study is to characterize soil fungi from sites exposed to different wood preservative types and to analyze differences in fungal species composition due to long-term preservative exposure. Simultaneously, the data generated will also serve as baseline data for continued monitoring of the soil fungal communities from different climates. A secondary objective of this study is to assess fungal diversity in copper exposed soils and compare to non-copper exposed soils to screen for presence of copper tolerant fungi.

## Materials and methods

### Field sampling

Field sites are located in Saucier, Mississippi and Madison, Wisconsin, USA. The Saucier site is located in the Harrison Experimental Forest in southern Mississippi. This geographic region is classified as high decay hazard for wood in service (zone 5-severe) according to both the American Wood Protection Association (AWPA) (Association, 2007) and the Scheffer index (Scheffer, 1971). This soil region is classified as coastal plain and the dominant soil type is sandy clay with surrounding overstory being predominantly loblolly pine (*Pinus taeda L*.). Untreated southern pine sapwood will typically decay to the point of structural failure after 1 year in ground exposure in this environment. The Saucier, Mississippi test site has been utilized by the United States Department of Agriculture Forest Service (USDA-FS) Forest Products Laboratory (FPL) as a preservative test site since 1940 and contains many of the early testing sites used to develop long-lasting and effective wood protectants. Samples were obtained on April 10, 2014 from 3 test plots at the field site. The mixed plot was a 50 year-old plot containing chromated copper arsenate (CCA), creosote and pentachlorophenol treated posts. The copper plot was a 30 year-old plot containing wooden stakes treated with basic solutions of copper salts. The control samples were obtained from a nearby, unused plot of forest within the Harrison Experimental Forest. Four field replicate soil samples per site-treatment combination were transported to the laboratory and frozen at −30°C for 4 days before processing.

The Madison, WI site has been used by FPL since 1950 for routine testing of experimental and existing preservative systems in a moderate decay hazard (AWPA, zone 2). The soil type at Madison is darker and contains more silt and clay as compared to the Saucier site and is subject to more freeze thaw cycles, in addition to a solid winter freeze. The Madison, WI site is mostly prairie/savannah type ecosystem with continuously decreasing overstory containing mostly mixed hardwoods [red maple (*Acer rubrum*), mixed oaks (*Quercus* spp.), and some black locust (*Robinia pseudoacacia*)]. In comparison to the Saucier site, untreated southern pine sapwood typically fails due to decay within 2 years at this more northern site. Samples were obtained on May 9, 2014 from 3 test plots at the Madison field site. The mixed plot was an approximately 60 year-old plot containing CCA, creosote and pentachlorophenol treated posts approximately 10 cm in diameter. The copper plot was a 40 year-old plot containing 60 cm (2 ft) × 120 cm (4 ft) wooden stakes treated with various copper solutions. The control samples were obtained from a nearby, unused plot of forest within the FPL test site approximately 1 km from the treated plots.

Samples were taken from the soil at both sites using 5 g Terra Core (En Novative Technologies, Dexter, MI, USA) disposable soil samplers. Samples were taken from four locations approximately 10 feet apart within each test plot and 3 plugs were combined within each treatment replicate. Four field replicate soil samples were transported to the laboratory and frozen at −30°C for 3 days before processing.

### DNA isolation and amplicon based sequencing

A 0.25 g aliquot of each hand mixed sample was extracted using the MoBio Power Soil DNA Isolation Kit (Carlsbad, CA, USA) following manufacturer's instructions. The samples were eluted in 100 μl C6 elution buffer and cleaned using the MoBio Powerclean Pro DNA Clean-up Kit following manufacturer's instructions. Samples were eluted in 100 μl DC5 elution buffer then quantified by spectrophotometer and diluted to 10 ng/μl in Tris EDTA pH 8. DNA samples were amplified in triplicate using 25 ng template or water controls and ITS1F and ITS2 primers (De Gannes et al., 2013) with Illumina adapters for the MiSeq platform with 24 unique identifiers on the reverse primers. Phusion Hot Start Flex DNA Polymerase (New England Biolabs, Ipswich, MA, USA) in HF buffer was used according to manufacturer's instructions for PCR with the following program: 4 min at 94°C, followed by 30 cycles of 30 s at 94°C, 60 s at 50°C and 90 s at 72°C and a final extension of 10 min at 72°C. Check gels were run on one of each PCR replicate and 400–500 bp products were confirmed. The three PCR replicates were combined and cleaned using Agencort AMPure XP beads following manufacturer instructions. Each sample was quantified using the Quant-it DNA Assay Kit (high sensitivity, ThermoFisher, Waltham, MA, USA) following the microplate procedure with a Synergy H1 multimodal plate reader (Biotek, Winooski, VT, USA). All samples were then normalized to 10 nM and combined in equal amounts. This pooled sample was submitted to the University of Wisconsin-Madison Biotechnology Center—DNA Sequencing Facility for 250 paired Illumina MiSeq sequencing using paired reads of 250 base pairs.

### Sequencing data analysis

Sequencing data were processed using the AMPtk v0.4.0 pipeline (Palmer, 2015 -https://github.com/nextgenusfs/amptk). Briefly, overlapping 2 × 250 base pair Illumina MiSeq reads were merged using USEARCH v8.1.1831 (Edgar and Flyvbjerg, 2015), forward and reverse primers were removed from the merged reads, and the reads were trimmed or padded with N's to a set length of 250 base pairs. Because ITS sequences are of variable length they require extra processing steps in comparison to 16S reads that are nearly identical in length. The average length of known ITS1 sequences in public databases is ~250 bp; ranging in size from ~150 bp up to >600 bp, thus we aren't able to recover ^*^all^*^ full length sequences with PE 2 × 250 bp sequencing. And it is recommended to use reads that are truncated to the same length when clustering with UPARSE pipeline, padding/trimming to 250 bp was used to maximize the number of reads passing the quality filters of AMPtk as well as provide enough information to assign taxonomy to OTUs. Processed reads were quality trimmed based on accumulation of expected errors less than 1.0 (Edgar and Flyvbjerg, 2015) and clustered using the UPARSE algorithm using default parameters (singletons removed, 97% OTU radius). An OTU table was generated by mapping the original reads to the OTUs using VSEARCH 1.9.1 (Rognes et al., 2016) and the OTU table was subsequently filtered to eliminate “index-bleed” between samples by setting read counts to zero if the number of reads mapped was less than 0.5% of the sum of all read counts for each OTU. Index-bleed (also called index-crossover, index-hopping, barcode-mismatch, etc.) is a phenomenon in NGS sequencing where a small number of reads are mis-assigned to the wrong sample group during multiplex sequencing (combining multiple samples on a single NGS sequencing run). Thus to filter out low read counts that could have arisen from sample mis-assignment (index-bleed), AMPtk provides a filter to clean an OTU table. Taxonomy was assigned using a combination of UTAX and global alignment (USEARCH Edgar, 2010) to the UNITE v7.0 database (Abarenkov et al., 2010) and non-fungal OTUs were removed prior to downstream data processing. BIOM data from AMPtk pipeline was further analyzed using METACOMET (Wang et al., 2016) and PHINCH (Bik and Pitch Interactive, 2014) to visually compare treatment and sites at each taxonomic level.

### Fungal community analyses

All distance-matrix based community analyses were performed on a presence absence (binary) OTU matrix, using the Raup-Crick distance metric as calculated by the raupcrick function in the vegan package (Oksanen et al., 2015) of R (R Core Team, 2013). This metric is robust to common issues in large data sets such as an abundance of zeros, and variation in alpha diversity among samples (Chase et al., 2011). To visualize fungal communities, we performed ordinations using non-parametric multi-dimensional scaling (NMDS), implemented by the metaMDS function in the vegan package (Oksanen et al., 2015) of R (Team, 2016). Permutational multivariate analysis of variance (PERMANOVA) tests were used to test for site and treatment effects. PERMANOVA was calculated by the Adonis function in the vegan package (Oksanen et al., 2015). We also tested for multivariate dispersion among groups using the betadisper function in the vegan package of R. Finally, we performed indicator species analyses to identify specific fungal OTUs associated with each treatment group at each site using the multipatt function in the indicspecies package (Cáceres and Legendre, 2009) in R.

### Inductively coupled plasma-optical emission spectroscopy (ICP-OES) methods

Twenty four soil samples were dried at 105°C for 2 days, then immediately capped and transferred to a desiccator. Samples were weighed in a dry room to 4 decimal places and directly into a tared Teflon digestion vessel-Anton Paar (Ashland, VA) HVT50 vessels. Two milliliters of 70% HNO_3_ (Sigma-Aldrich (Milwaukee, WI) ACS-grade) was added, using an acid-resistant bottle-top pipette, to each digestion vessel. Samples were pre-digested for 15 min. at room temperature. In a fume hood, 5 ml 18 MΩ H_2_O was added, and further digested for 10 min, then vessels were sealed. Digested solutions were cooled, then filtered through glass microfiber filter (Whatman 934-AH) into a 50-ml volumetric flask and brought to volume using 18 MΩ H_2_O.

A Horiba (Edison, NJ) ULTIMA II high resolution spectrometer was used for the ICP-OES analysis. The instrument was equipped with a solid state, water cooled 40 MHz radio frequency source, a Czerny Turner monochrometer with 1 meter focal length, nitrogen purged optical bench at 6 L/min, holographic grating (2400 grooves/mm) with resolution <5 pm in the 120–320 nm range (1st order) and <10 pm in the 320–800 nm range (2nd order), and a vertical torch with radial viewing. Power level was 1,000 W. Gas flows were: Plasma gas 12 L/min, sheath gas 0.2 L/min, nebulizer gas 0.28 L/min. Sample introduction used a Conical U-series concentric glass nebulizer (1 ml/min) and a Tracey spray chamber with helix connection and TruFlow sample monitor (Glass Expansion, Pocasset, MA).

Standards were prepared from 1,000 ppm stock solutions without serial dilution. Standard curves were obtained using external standards for Ca 396.847, Ca 422.673, Cr 205.552, Cr 267.716, Cu 224.700, Cu 324.750, Cu 327.396, K 766.455, Mg 279.079, Mg 280.270, Mg 285.213, and P178.229 nm. Each standard curve was checked using a separate calibration solution (Inorganic Ventures (Christiansburg, VA) ICP-MS 71A multi-element standard), then the best line chosen for quantitation of samples. The calibrations, each with a correlation coefficient *r*^2^ > 0.9999, were checked at the start, middle and end of the ICP sequence, with pass falling within 10% of target. Ten replicate readings of a blank were also run at the start, middle and end of the ICP sequence, then used to determine Limit of Quantitation (LOQ). Acquisition was done in Max mode peak shape, with 0.5 s integration time and 3 replicate readings averaged for the reported measurement. A 20/15 um slit combination was used for all lines. The instrument electronics were turned on ~30 min. prior to start in order to minimize instrumental drift. Prior to each measurement, the sample intake was rinsed 20 s with 5% HNO3 (v/v) at high pump speed, followed by a 20 s rinse with analyte solution at high pump speed, then a 20 s plasma equilibration period at normal pump speed. Prior to calibration, a profile acquisition was taken to visually inspect peak shape and manually set background correction points at peak base.

## Results

A total of 6,663 OTUs were recovered from the 24 soil samples. Thirty-one protist OTUs were omitted from the data set. A total of 3,110 OTUs were identified only to the Kingdom level and 3,448 OTUs were classified to the phylum level. A total of 1,881 OTUs were classified as Ascomycetes (Phylum = Ascomycota), 1,040 OTUs were classified as Basidiomycetes (Phylum = Basidiomycota), 147 were classified as Zygomycetes (Phylum = Zygomycota) and 233 were classified as Glomeromcyetes (Phylum = Glomeromycota), 4 OTUS were classified as Blastocladiomycota, which are recently split from the Chytridomycota (James et al., 2006), 78 OTUs were classified as Chytridomycota, and 55 OTUs were classified as Rozellomycota, which represent another lineage recently split from the Chytridomycota, These are typically amoeboid microfungi and are almost exclusively identified through environmental sequencing. The distribution of the different phyla recovered from the samples is shown in Supplemental Figure 1.

A total of 25 fungal classes were identified from the soil samples. The number of representative OTUs of the class Archeorhizomycetes was noticeably higher in several of the preservative exposed samples. In addition to several unidentified classes, representatives of class Agaricomycetes, Archeorhizomycetes, Sordariomycetes, Eurotiomyctes, Leotiomycetes, and Dothidiomycetes were all abundant in our sampling. Their relative abundances and distributions across the samples are shown in Supplemental Figure 2.

A total of 55 fungal orders were classified. Order Agaricales was abundant in our sampling and widespread across both site and treatment. Order Mortierellales was also abundant and widespread, although more abundant at the MS site. Order Russulales exhibited a patchy distribution and was found in unexposed, mixed treatments and copper treated plots intermittently and much less prevalent at the WI site. Both within the class Sordariomycetidae, Order Hypocreales was more abundant in MS while order Sordariales was more prevalent in WI. Both of these classes contain ascomycete fungi typically associated with soft rot as well as several important plant pathogens. Order Thelophorales was more prevalent in MS but did not appear to be affected by treatment. Relative abundances and distributions of the orders are shown in Supplemental Figure 3.

A total of 206 fungal families were classified. The 10 most common families were Mortierrellaceae, Hygrophoraceae, Russullaceae, Clavariaceae, Tricholomataceae, and Thelephoraceae with four additional unidentified families. Representatives of the Hygrophoraceae were sparsely distributed but more prevalent in WI. Family Russulaceae was more prevalent in the MS unexposed areas. Family Tricholomataceae was sparsely distributed among mixed preservative and copper treated sites in MS. Family Clavariaceae was sparsely distributed in WI among all the treatments and less common in MS. Class Archeorhizomycetes was more prevalent in the preservative exposed sites and far less common in the unexposed areas. Family Mortierrellaceae was highly abundant and widespread in MS and widespread but less abundant in WI. Relative abundances and distributions of the Families are shown in Supplemental Figure 4.

A total of 1,136 OTUs were classified to the genus level with an additional 509 OTUs classified to species. The genus *Mortierella* was highly abundant and widespread in MS but also widespread in WI with lower abundance. Four additional fungal unidentified genera were sparsely distributed. The genus *Hygrocybe* was more abundant in the WI soil samples especially one unexposed sample. The genus *Lactarius* was widely distributed and found in both sites (MS and WI). Relative abundances and distributions of the genera are shown in Supplemental Figure 5.

*Mortierella humulis* was the only widespread, abundant fungus identified to species in the study and was highly prevalent in MS, but sparsely distributed in WI.

### Fungal community analyses

In order to compare fungal species composition between sites and treatments, PERMANOVA was used to analyze the taxonomy data. Due to large differences in community composition between the MS and WI sites, data sets were analyzed separately.

**Figure 1:**
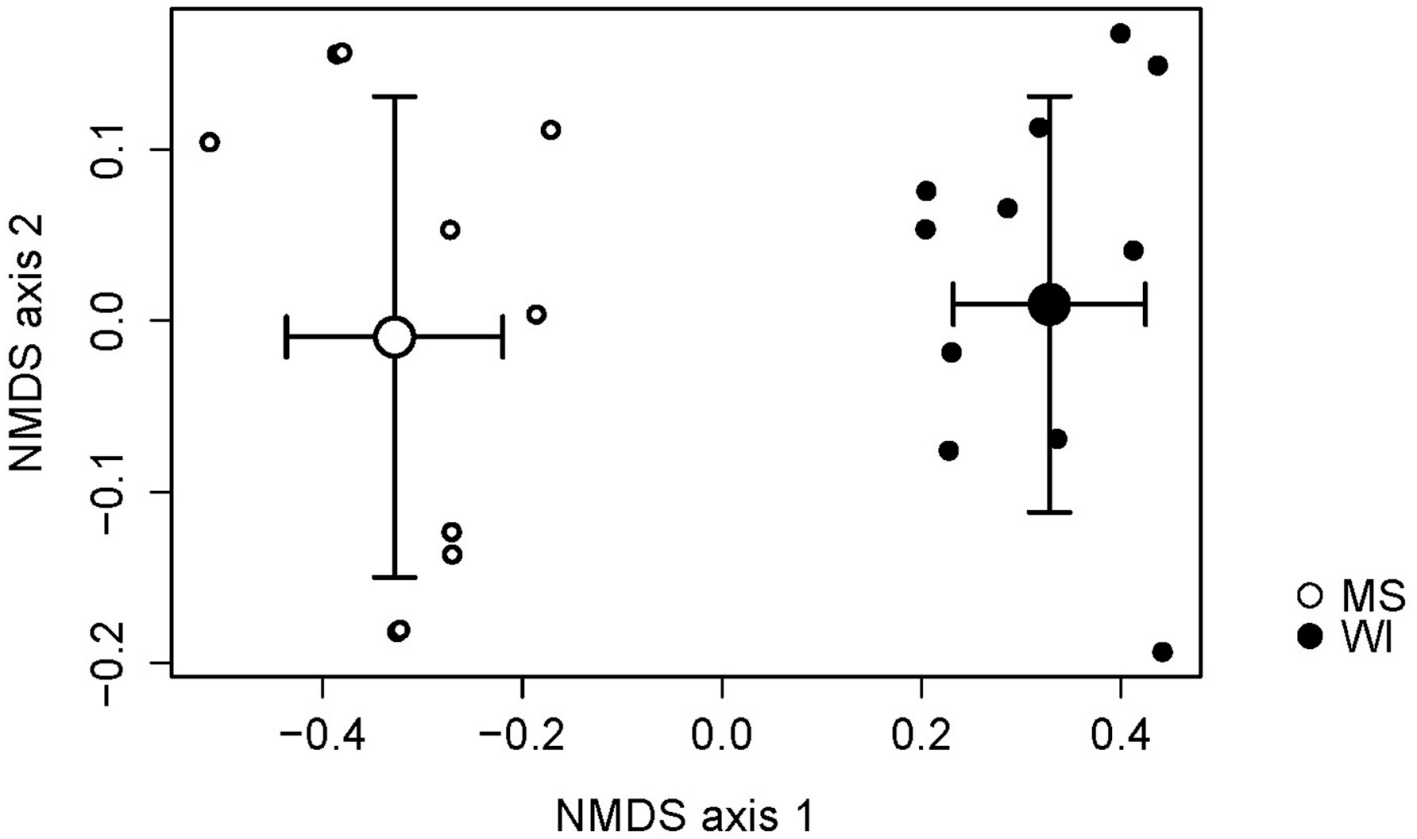
Non-metric multidimensional scaling (NMDS) plot indicating the effects of site on the fungal species composition. Sites were distinctly different and necessitated separate analyses of treatments.

Sites were significantly different from each other with respect to fungal species composition (*P* < 0.0001) (Figure 1). Chemical analysis showed richer nutrient profiles in the MS site and lower pH of soil (Table 1). Highly correlated OTUs that contributed to the differences between sites along each NMDS axis were compiled in PERMANOVA and the five OTUs with the highest and lowest degrees of correlation for each site and axis of the NMDS ordination are listed in Table 2. These represent outliers from the shared core fungal groups within the total pool of OTUs and help to define differences between the sites.

**Table 1:** Results of chemical analysis of soil samples.

**SamplelD**	**Sample#**	**Site**	**Treat**	**Rep**	**Descr**	**Lat**	**Long**	**pH**	**Ca**	**Cr**	**Cu**	**K**	**Mg**	**P**	**As**
WIA1	1	WI	A	1	Mixed	43°02′39″N	89°33′18″W	7	2.88	0.02	0.03	1.60	2.94	0.55	0.01
WIA2	2	WI	A	2	Mixed	43°02′39″N	89°33′18″W	6.15	2.44	0.02	0.03	1.56	2.85	0.55	0.01
WIA3	3	WI	A	3	Mixed	43°02′39″N	89°33′18″W	6.82	2.52	0.02	0.03	1.74	3.20	0.57	0.01
WIA4	4	WI	A	4	Mixed	43°02′39″N	89°33′18″W	6.23	2.76	0.01	0.03	1.32	2.62	0.56	0.01
WIB1	5	WI	B	1	Cu	43°02′40″N	89°33′09″W	6.18	3.32	0.01	0.03	1.52	2.54	0.64	0.01
W IB2	6	WI	B	2	Cu	43°02′40″N	89°33′09?W	6.15	2.55	0.01	0.03	1.33	2.59	0.57	0.01
WIB3	7	WI	B	3	Cu	43°02′40″N	89°33′09″W	6.09	3.36	0.01	0.03	1.16	3.01	0.48	0.01
WIB4	8	WI	B	4	Cu	43°02′40″N	89°33′09″W	6.5	2.84	0.01	0.03	1.34	2.57	0.43	0.01
WIC1	9	WI	C	1	Control	43°02′40″N	89°33′04″W	6.85	99.89	0.00	0.02	0.76	54.12	0.67	nd
WIC2	10	WI	C	2	Control	43°02′40″N	89°33′04″W	6.8	101.35	0.00	0.02	0.71	54.66	0.78	nd
WIC3	11	WI	C	3	Control	43°02′40″N	89°33′04″W	6.47	2.91	0.02	0.03	1.48	3.02	0.58	0.01
WIC4	12	WI	C	4	Control	43°02′40″N	89°33′04″W	6.3	2.89	0.02	0.03	1.77	2.85	0.65	0.01
MSA1	13	MS	A	1	Mixed	30°37′44″N	89°02′46″W	4.59	0.89	0.01	0.01	0.57	0.59	0.05	0.01
MSA2	14	MS	A	2	Mixed	30°37′44″N	89°02′46″W	4.95	0.25	0.01	0.01	0.23	0.29	0.12	0.00
MSA3	15	MS	A	3	Mixed	30°37′44″N	89°02′46″W	4.99	2.68	0.00	0.01	0.58	0.51	0.24	0.01
MSA4	16	MS	A	4	Mixed	30°37′44″N	89°02′46″W	4.94	0.38	0.00	0.01	0.28	0.30	0.12	0.02
MSB1	17	MS	B	1	Cu	30°37′44″N	89°02′47″W	4.82	0.36	0.00	0.01	0.21	0.26	0.11	nd
MSB2	18	MS	B	2	Cu	30°37′44″N	89°02′47″W	5.22	0.40	0.00	0.01	0.16	0.20	0.08	0.00
MSB3	19	MS	B	3	Cu	30°37′44″N	89°02′47″W	4.97	1.48	0.00	0.33	0.37	0.45	0.29	nd
MSB4	20	MS	B	4	Cu	30°37′44″N	89°02′47″W	5.16	0.09	0.00	0.02	0.18	0.24	0.07	nd
MSC1	21	MS	C	1	Control	30°37′42″N	89°33′04″W	4.83	0.64	0.00	0.01	0.28	0.31	0.25	0.00
MSC2	22	MS	C	2	Control	30°37′42″N	89°33′04″W	5.33	0.24	0.00	0.01	0.21	0.20	0.15	nd
MSC3	23	MS	C	3	Control	30°37′42″N	89°33′04″W	5.14	0.27	0.01	0.01	0.30	0.30	0.22	0.00
MSC4	24	MS	C	4	Control	30°37′42″N	89°33′04″W	5.08	0.60	0.00	0.01	0.28	0.28	0.18	0.00

*pH was determined by benchtop pH meter and soil elemental concentrations were determined by ICP-OES and are expressed in mg/kg of soil. WI soils were richer in macro-nutrients and also with higher pH values than those in MS. Residual preservatives were not elevated in any of the plots compared to untreated areas with only one exception (MSB3-elevated copper)*.

**Table 2:** Highly correlated OTUs contributing to differences between sites MS and WI.

**OTU**	**Corr**	**Taxonomy**	**OTU**	**Corr**	**Taxonomy**
**MS NMDS-axis 1 top 5 positively correlated OTUS**			**WI NMDS-axis 1-top 5 positively correlated OTUs**		
OTU_357	0.91972	*Phialocephala humicola*	OTU_66	0.961668	*Mortierella humilis*
OTU_1091	0.91972	*Wickerhamomyces subpelliculosus*	OTU_99	0.961668	*Saccharomycopsis crataegensis*
OTU_1476	0.871663	*Sebacinales*	OTU_148	0.961668	*Penicillium copticola*
OTU_1531	0.871663	*Glomus proliferum*	OTU_199	0.961668	*Mortierella* spp.
OTU_2139	0.871663	*Tomentella amyloapiculata*	OTU_284	0.961668	*Kodamaea ohmeri*
**MS NMDS-axis 1 top 5 negatively correlated OTUS**			**WI NMDS-axis 1-top 5 negatively correlated OTUs**		
OTU_644	−0.88078	*Pseudozyma prolifica*	OTU_6907	−0.96167	*Tremella subalpina*
OTU_3456	−0.88078	*Genea cephalonicae*	OTU_7079	−0.96167	*Batcheloromyces sedgefieldii*
OTU_523	−0.91972	*Hygrocybe virginea*	OTU_7086	−0.96167	*Clydaea vesicula*
OTU_876	−0.91972	*Anthostomella leucospermi*	OTU_7117	−0.96167	*Mycosphaerella elongata*
OTU_1781	−0.91972	*Cladophialophora chaetospira*	OTU_7260	−0.96167	Unidentified
**MS NMDS-axis 2 top 5 positively correlated OTUS**			**WI NMDS-axis 2-top 5 positively correlated OTUs**		
OTU_570	0.803391	*Glomus proliferum*	OTU_135	0.89658	*Populocrescentia forlicesenensis*
OTU_950	0.803391	*Claroideoglomus luteum*	OTU_994	0.851367	*Buckleyzyma aurantiaca*
OTU_2713	0.784669	*Resinicium furfuraceum*	OTU_3609	0.851367	*Scytalidium lignicola*
OTU_4984	0.751979	*Ceramothyrium podocarpi*	OTU_1506	0.766713	*Clavaria falcata*
OTU_145	0.705732	*Umbelopsis swartii*	OTU_2178	0.766713	*Glomus proliferum*
**MS NMDS-axis 2 top 5 negatively correlated OTUS**			**WI NMDS-axis 2-top 5 negatively correlated OTUs**		
OTU_2638	−0.87761	*Protrudomyces lateralis*	OTU_128	−0.66493	*Mortierella hyalina*
OTU_246	−0.88503	*Mortierella echinula*	OTU_816	−0.66493	*Hebeloma erebium*
OTU_568	−0.88503	*Umbilicaria iberica*	OTU_3820	−0.66493	*Lactifluus subiculatus*
OTU_1984	−0.88503	*Periconia macrospinosa*	OTU_349	−0.74489	*Polyscytalum algarvense*
OTU_2630	−0.88503	*Tremella samoensis*	OTU_546	−0.74489	*Hirsutella rhossiliensis*

*i.e., Core groups of fungal species that can be used to distinguish between the two sites*.

**Figure 2:**
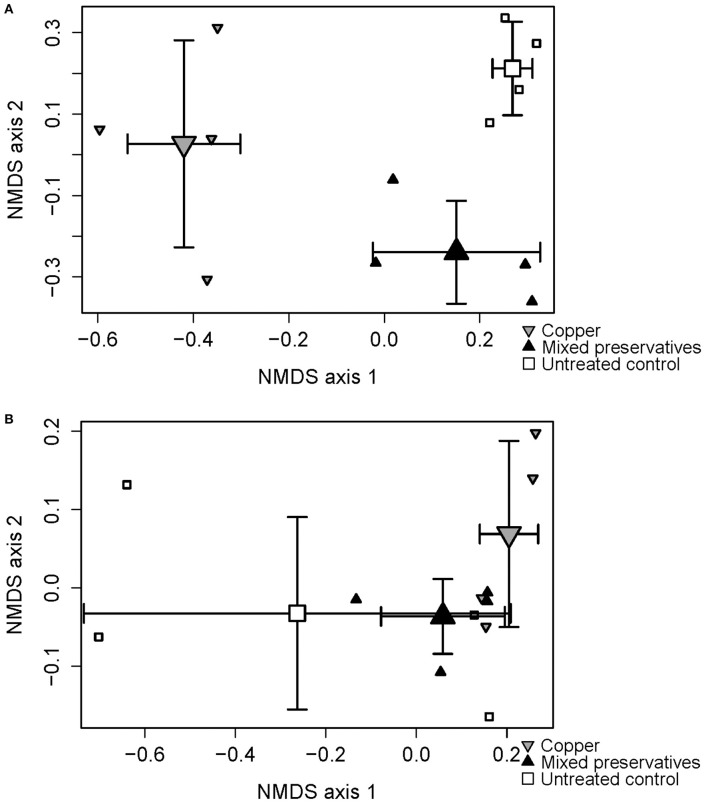
Non-metric multidimensional scaling (NMDS) ordination plot displaying differences between treatments with respect to fungal species composition at **(A)** MS test site and **(B)** WI test site.

Significant treatment and dispersion effects were seen in MS (PERMANOVA on treatment r-squared = 0.96, *p* < 0.0001, *F* = 125 Mississippi betadisper *F* = 61.871 p < 0.001) and WI (PERMANOVA on treatment r-squared = 0.39, *p* = 0.09, *F* = 2.85 betadisper *F* = 7.98, *p* < 0.001). 2-Dimensional results from the Non-metric Multidimensional Scaling (NMDS) analysis are presented in Figure 2 showing treatment differences in both MS (Figure 2A) and WI (Figure 2B).

### Indicator species analysis

Thirteen indicator species were found in the mixed preservative MS plot (Table 3); of those, 6 were highly correlated (corr Val = 1). Twenty-three indicator species were found in the mixed preservative WI plot (Table 4); of those, 8 were highly correlated (corr Val = 1). A total of 14 indicator species were identified with the copper treatments in MS (Table 5), three of which were highly correlated with copper treatments (Corr_val = 1) and the remainder were still correlated, but to a lesser extent (Corr_val = 0.894). A total of 7 indicator species were identified for copper exposed soils in WI (Table 6). Two species (*Orbillia* sp. and *Rhytidhysteron rufulum*) had the highest degree of association (Corr_val = 1), while the remaining species were less correlated (Corr_value = 0.894–0.0866). Twenty-five indicator species were found in the unexposed MS plot (Table 7); of those, 6 were highly correlated (corr Val = 1). A total of four indicator species were identified for the WI unexposed soil (Table 8).

**Table 3:** Indicator species for soil exposed to mixed preservative chemistries in MS for 50 years.

**OTU_ID**	**Corr_val**	***p*-value**	**Species_ID**
OTU_46	1	0.010^**^	*Cenococcum geophilum*
OTU_416	1	0.010^**^	*Herpotrichiellaceae* sp.
OTU_463	1	0.010^**^	*Ceriporiopsis* sp.
OTU_1356	1	0.010^**^	*Neofusicoccum grevillea*
OTU_2038	1	0.010^**^	*Orbilia* sp.
OTU_2783	1	0.010^**^	*Neopaxillus dominicanus*
OTU_246	0.894	0.034^*^	*Mortierella echinula*
OTU_568	0.894	0.034^*^	*Pleosporales* sp.
OTU_1223	0.894	0.034^*^	*Ramariopsis corniculata*
OTU_1299	0.894	0.035^*^	*Ramariopsis* sp.
OTU_1980	0.894	0.031^*^	*Botryobasidium* sp.
OTU_1984	0.894	0.034^*^	*Periconia macrospinosa*
OTU_2630	0.894	0.034^*^	*Tremella samoensis*

* Indicates significance at the 0.05 level, and

** *indicates significance at the 0.005 level*.

**Table 4:** Indicator species for soil exposed to mixed preservative chemistries in WI for 60 years.

**OTU_ID**	**Corr_val**	***p*-value**	**Species_ID**
OTU_55	1	0.006^**^	*Clavicorona taxophila*
OTU_378	1	0.006^**^	*Nothojafnea cryptotricha*
OTU_578	1	0.006^**^	*Archaeorhizomyces borealis*
OTU_1242	1	0.006^**^	*Ganoderma lucidum*
OTU_1288	1	0.006^**^	*Podospora intestinacea*
OTU_1882	1	0.006^**^	*Flagelloscypha citrispora*
OTU_2588	1	0.006^**^	*Coniothyrium* sp.
OTU_3787	1	0.006^**^	*Operculomyces laminatus*
OTU_282	0.894	0.036^*^	*Inocybe* sp.
OTU_807	0.894	0.027^*^	*Fellomyces lichenicola*
OTU_905	0.894	0.027^*^	*Lactarius chrysorrheus*
OTU_931	0.894	0.029^*^	*Oliveonia pauxilla*
OTU_1118	0.894	0.029^*^	*Glomus* sp.
OTU_1382	0.894	0.029^*^	*Lophiostoma corticola*
OTU_1442	0.894	0.029^*^	*Myrothecium leucotrichum*
OTU_1938	0.894	0.026^*^	*Penicillium coprobium*
OTU_3470	0.894	0.026^*^	*Kodamaea ohmeri*
OTU_18	0.866	0.048^*^	*Ramariopsis helvola*
OTU_1226	0.866	0.050^*^	*Lasiodiplodia crassispora*
OTU_1639	0.866	0.050^*^	*Sarocladium mycophilum*
OTU_2694	0.866	0.048^*^	*Mortierellales* sp.
OTU_2801	0.866	0.048^*^	*Pachyphlodes citrinus*
OTU_3788	0.866	0.048^*^	*Cladophialophora* sp.

* Indicates significance at the 0.05 level, and

** *indicates significance at the 0.005 level*.

**Table 5:** Indicator species for MS for soils exposed to copper treatments for ~30 years.

**OTU_ID**	**Corr_val**	***P*-value**	**BLAST result**
OTU_523	1	0.009^**^	*Hygrocybe virginea* var. *ochraceopallida*
OTU_876	1	0.009^**^	*Anthostomella leucospermi*
OTU_1781	1	0.009^**^	*Cladophialophora chaetospira*
OTU_95	0.894	0.045^*^	*Cladophialophora* sp.
OTU_521	0.894	0.029^*^	*Helotiales* sp.
OTU_644	0.894	0.031^*^	*Pseudozyma prolifica*
OTU_898	0.894	0.029^*^	Dothidiomycetes sp.
OTU_996	0.894	0.045^*^	*Phialophora europaea*
OTU_1669	0.894	0.045^*^	*Sordariales* sp.
OTU_1981	0.894	0.036^*^	*Leccinum talamancae*
OTU_2235	0.894	0.038^*^	*Cladophilaophora* sp.
OTU_1390	0.866	0.047^*^	*Dactylaria* sp.
OTU_2283	0.866	0.047^*^	*Glomus diaphanum*
OTU_4748	0.866	0.047^*^	*Tremellodendropsis tuberosa*

* Indicates significance at the 0.05 level, and

** *indicates significance at the 0.005 level*.

**Table 6:** Indicator species for WI soils exposed to copper treatments for 40 years.

**OTU_ID**	**Corr_val**	***p*-value**	**Species ID**
OTU_332	1	0.007^**^	*Orbilia* sp.
OTU_3106	1	0.007^**^	*Rhytidhysteron rufulum*
OTU_472	0.894	0.026^*^	*Tuber* sp.
OTU_1506	0.866	0.046^*^	*Clavaria falcate*
OTU_2178	0.866	0.046^*^	*Glomus* mycorrhizal symbiont of Marchantia foliacea
OTU_2190	0.866	0.046^*^	*Glomus* sp.
OTU_2313	0.866	0.046^*^	*Metarhizium anisopliae*

* Indicates significance at the 0.05 level, and

** *indicates significance at the 0.005 level*.

**Table 7:** Indicator species for unexposed soils in MS.

**OTU_ID**	**Corr_val**	***p*-value**	**Species_ID**
OTU_330	1	0.008^**^	Ericoid mycorrhizal sp.
OTU_1273	1	0.008^**^	*Arthrinium arundinis*
OTU_1618	1	0.008^**^	*Cryptococcus cuniculi*
OTU_2246	1	0.008^**^	*Oliveonia pauxilla*
OTU_2485	1	0.008^**^	*Craterocolla cerasi*
OTU_3377	1	0.008^**^	*Flagelloscypha citrispora*
OTU_20	0.894	0.036^*^	*Lactarius cf. subserifluus*
OTU_412	0.894	0.033^*^	*Cladophialophora* sp.
OTU_672	0.894	0.042^*^	*Cladophialophora* sp.
OTU_688	0.894	0.042^*^	*Dioszegia rishiriensis*
OTU_1226	0.894	0.031^*^	*Lasiodiplodia crassispora*
OTU_1447	0.894	0.033^*^	*Mucoromycotina* sp.
OTU_1515	0.894	0.042^*^	*Limonomyces culmigenus*
OTU_7398	0.894	0.036^*^	*Herpotrichiellaceae* sp.
OTU_131	0.866	0.049^*^	*Cenococcum geophilum*
OTU_339	0.866	0.049^*^	*Scleroderma polyrhizum*
OTU_462	0.866	0.049^*^	*Lambiella caeca*
OTU_700	0.866	0.049^*^	*Hyaloscyphaceae* sp.
OTU_1089	0.866	0.049^*^	Glomeromycetes sp.
OTU_1102	0.866	0.049^*^	*Pulvinula constellation*
OTU_1564	0.866	0.049^*^	Dothideomycetes sp.
OTU_2047	0.866	0.049^*^	*Glomus* sp.
OTU_2629	0.866	0.049^*^	*Mortierellales* sp.

* Indicates significance at the 0.05 level, and

** *indicates significance at the 0.005 level*.

**Table 8:** Indicator species for unexposed soils in WI.

**OTU_ID**	**Corr_val**	***p*-value**	**Species_ID**
OTU_372	0.866	0.049^*^	*Coniochaetales* sp.
OTU_928	0.866	0.049^*^	*Septoglomus* sp.
OTU_1201	0.866	0.049^*^	*Geotrichum candidum*
OTU_3016	0.866	0.049^*^	*Ophiostoma piliferum*

* *Indicates significance at the 0.05 level*.

### Functional guilds analysis

Of the total 6,668 OTUs, 1,330 (20%) of the OTUs were classified using Funguild (Nguyen et al., 2016), which assigns functional guild information to taxonomic sequence data.

## Discussion

This study marks our first amplicon-based DNA sequence analysis of soil fungi under long-term wood preservative exposure. Characterization of soil fungi by metabarcode analysis gives a prediction of which fungi are present in a given location and can give highly detailed information about the decay potential of a given site. This study provided baseline data about our field sites that can be used to assist in future field exposure decisions.

### Community analysis

Unsurprisingly, the fungal soil communities differed between our two field sites that were separated by 995 miles and 13° of latitude. Overall, a greater number of OTUs were found in the WI site, presumably due to the more neutral soil pH and richer soil nutrient composition (see Table 1). Differences between treatments were also noted for both sites with greater difference between treatments in the harsher decay hazard climate (MS); treatments were also different in WI but with much greater variability.

### Indicator species

Indicator species were determined for each site treatment combination and notable observations were the low diversity of wood saprobes present in the soil, which did not appear to be influenced by treatment history but did differ between test sites. Several ectomycorrhizal fungi were found to be associated with both mixed and Cu preservatives exposure, which agrees with previous literature (Meharg and Cairney, 2000; Fomina et al., 2005; Colpaert, 2008), suggesting that ectomycorrhizal fungi are able to persist and might be beneficial remediators for preservative exposed soils, and that long-term preservative exposure has little effect on the abundance and prevalence of this group of fungi.

Thirteen indicator species were found in the mixed preservative MS plot (Table 2); of those, 6 were highly correlated (corr Val = 1). *Cenococcum geophilum* is a mycorrhizal species associated with a diverse list of hosts with wide distribution and is routinely identified from soil, based on morphology of colonized roots (Douhan and Rizzo, 2005). Herpotrichiellaceae is a family of loculoascomycetes with black yeast anamorphs (Untereiner et al., 1995). *Ceriporiopsis* sp. include lignin degrading white-rot fungi studied for enzymes that can be used in biopulping (Ferraz et al., 2003). *Neofusicoccum grevillea* is a pathogen on *Frivillea aurea* causing a leaf spot (Sakalidis et al., 2013). The genus *Neopaxillus* is in the crepidotaceae family in the agaricales, *N. dominicanus* is a new species found in the Dominican Republic in 2011 (Vizzini et al., 2012).

Twenty-three indicator species were found in the mixed preservative WI plot (Table 3); of those, 8 were highly correlated (corr Val = 1). Of the highly correlated, *Clavicorona taxophila* is a saprotroph in the Clavariaceae family including mushroom-forming fungi with *Clavicorona* producing coralloid sporocarps (Birkebak et al., 2013). It is a small coral fungus that is currently red-listed in the UK as threatened (Evans et al., 2006). *Nothojafnea cryptotricha* is a mycorrhiza of eucalypts (Warcup, 1990). *Archaeorhizomyces borealis* is a pine associated species of the soil-inhabiting genus commonly isolated from environmental samples (Menkis et al., 2014). They constitute a significant component of the rhizosphere in fungal DNA community analyses, comprising up to one third of the total fungal community (Porter et al., 2008). *Ganoderma lucidum* belongs to a genus of white rot fungi that are considered one of the most important medicinal fungi worldwide and is an ingredient in many health products supposedly having anti-cancer, anti-aging, and antimicrobial functions (Paterson, 2006). *Podospora intestinacea* is a coprophilous fungus commonly isolated form dung and produces a perithecial resting structure that can persist in soil or dung (Watling and Richardson, 2010). *Flagelloscypha citrispora* is a basidiomycete that occurs on rotten logs and stumps (Reid, 1964). Species of the *Coniothyrium* genus have been used as biocontrol for *Sclerotinia* as they are mycoparasites of mycelium and sclerotia; this treatment has been used in sunflower production (Whipps and Gerlagh, 1992). *Operculomyces laminatus* is a chytrid formerly known as *Rhizophlyctis harderi* (Powell et al., 2011).

A total of 14 indicator species were identified with the copper exposure in MS (Table 4). The highest correlations between species and copper treatment were for *Hygrocybe virginiana, Anthostomella leucospermi*, and *Cladophialophora chaetospira*.

A total of 7 indicator species were identified for copper exposed soils in WI (Table 5). *Orbillia* sp. is an operculate ascomycete belonging to the family Orbilliaceae which are often nematophagous (Ahrén et al., 1998); members of this genus have been isolated from forest soils in Argentina (Allegrucci et al., 2009). It produces an orange cup shaped fruiting body and has been observed on copper treated field stakes from our Wisconsin field site. Efforts are currently underway to confirm these fruiting bodies as being *Orbillia* spp. *Rhytidhysteron rufulum* is a saprophyte or weak parasite on a variety of plants most commonly associated with pan-tropical environments (Murillo et al., 2009).

Twenty-five indicator species were found in the unexposed MS plot (Table 6); of those, 6 were highly correlated (corr Val = 1). *Arthrinium arundinis* is a plant pathogen reported to cause kernel blight of barley (Crous and Groenewald, 2013). *Cryptococcus cuniculi* was previously described as an anamorphic yeast that was isolated from rabbit feces and is now considered a basidiomycetous yeast (Findley et al., 2009). *Oliveonia pauxilla* is a holobasidiomycete fungus and a saprobe on dead leaf litter (Warcup and Talbot, 1962). *Craterocolla cerasi* is a non-culturable fungus in the sebacinaceae group of the Hymenomycetidae family with fungi involved in various mycorrhizal associations (Weiss et al., 2004). *Flagelloscypha citrispora* is a wood saprobe also found in the mixed preservative plot in WI described above.

A total of four indicator species were determined for the WI unexposed soil (Table 7). A group containing Neotropical ascomycetes (*Coniochaetales* sp.) was correlated (0.866), and has a related species, *Coniochaetales lignaria*, that has been evaluated for its ability to break down furans and other phenolics that are the result of lignocellulose pretreatment for hydrolysis (López et al., 2004). *Septoglomus* sp. was also found to be correlated with unexposed soils in WI. The *Septoglomus* genus contains mostly mycorrhizal species that are widely distributed (Rydlová et al., 2015). This isolate clustered with *Septoglomus constrictum*, which has been isolated in other studies associated with heavy metal contaminated mine spoil (Shetty et al., 1994). *Geotrichum candidum* was also correlated (0.866) with untreated WI soils and has been widely studied for its production of lipases (Sugihara et al., 1990), phenol oxidases (Assas et al., 2000) and a host of other enzymes that can effectively break down a wide range of chemical classes (Sun et al., 2008). *Ophiostoma piliferum* is a sapstain fungus associated with bark beetles (Klepzig, 1998). The overall low diversity of the unexposed WI soils could be attributed to habitat. The samples were taken from a grassy prairie area with virtually no canopy and very little coarse woody debris—a driver of fungal diversity (Zellweger et al., 2015)—in the vicinity.

### Funguild analysis

**Figure 3:**
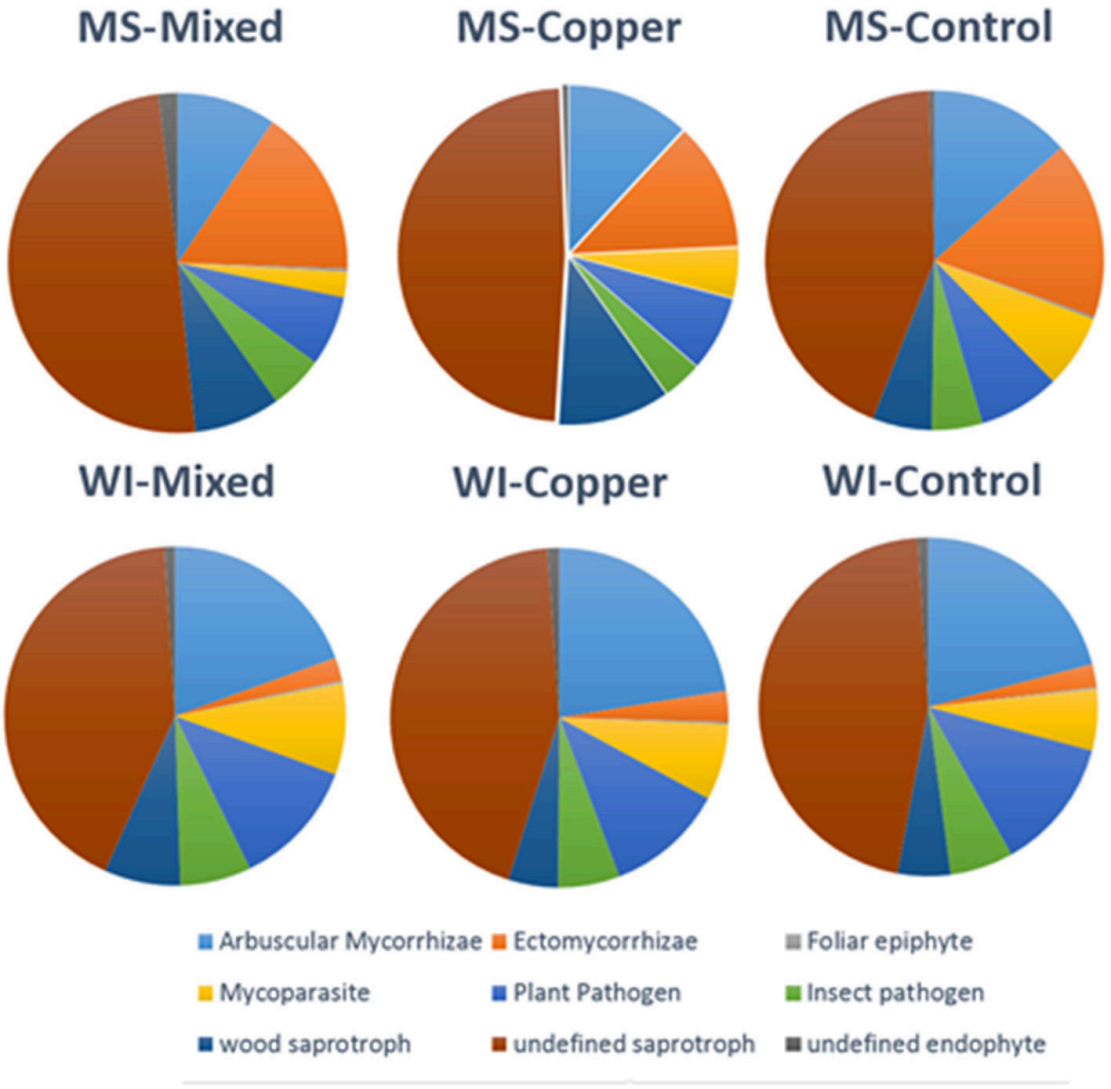
Distribution of major fungal guilds by site and preservative exposure of all OTUs classified using Funguild (*N* = 1,330 out of 6,668).

Relative proportions of fungal guilds were generally conserved across sites and preservative exposures. Pie charts showing proportions of guilds classified for each treatment group are shown in Figure 3. Wood saprobes compromised a relatively small portion of the total fungi detected in each sample group, likely owing to the fact that isolations where made from soil and not wood. Higher proportions of ECM fungi were noted at the MS site, but this is likely due to the predominant pine overstory which is heavily dependent on ECM fungi for growth and survival (Svenson et al., 1991) and predominately lower pH soils.

Analysis at the functional guild level showed that wood saprobes also make up a relatively small proportion of the total soil fungal community (7.29% of the OTUs characterized) and that saprophytic fungi, many of which are capable of inducing soft rot fungal degradation patterns in wood, comprise a large portion. A total of 67 OTUs were classified as animal pathogens, which include *Cryptococcus, Beauvaria, Metarhizium, Fusarium, Cordyceps*, and others. Two additional OTUs were classified as animal symbionts, both belonging to the genus *Rhodotorula*. A total of 233 OTUs were classified as Arbuscular Mycorrhizae mostly contained in the genus *Glomus*. A total of 41 OTUs were classified as dung associated saprotrophs mostly *Penicillium, Chaetomium*, or *Podospora*.

A total of 147 OTUs were classified as Ectomycorrhizal fungi and included the genera *Lactarius, Russula, Amanita, Tuber* and several other commonly found fleshy terrestrial fungi. Several representatives within this group were found to be indicator species in the community analysis, *Lactarius chrysorrheus*-(mixed preservative associated in WI), *Tuber* sp. (Cu associated in WI), and *Leccinum talamanaceae* (Cu associated in MS). Ectomycorrhizal fungi have been found to possess abilities to biotransform and sequester toxins, such as arsenic in the form of arsenobetaine (Nearing et al., 2015) and other persistent heavy metals contaminating soil (Khan et al., 2000). Previous works to bio-augment and remediate treated wood waste and soil have revolved primarily around preservative tolerant species of polyporoid fungi (De Groot and Woodward, 1999; Illman and Yang, 2004) and have not fully explored the roles of these terrestrial fungi as potential remediators. Ectomycorrhizal fungi have been studied for their role in remediating heavy metal contaminated soil and several species that are known to be metal tolerant were detected in our study (*Cenococcum geophilum, Amanita* spp., and *Pisolothus tinctorus*). An important exception would be *H. virginiana*, which was found to be highly associated with Cu treatments in MS, but is actually classified as an undefined saprotroph since *Hygrocybe* species are considered to be neither saprotrophic nor mycorrhizal (Seitzman et al., 2010) but symbionts of mosses. A total of 17 OTUs were classified as endophytes and included many common plant pathogens (*Phialophora, Cladosporium, Periconia macrospinosa*). Representatives of this guild were found as indicator species in both unexposed (*Herpotrichiellaceae* sp.), mixed preservative exposed (*P. macrospinosa*) and Cu exposed soils (*Phialophora europaea*). A total of 590 OTUs were classified as saprophytes and included an extremely wide range of species from the entire spectrum of fungal taxonomy (lower fungi, such as Mortierella to higher basidiomycetes, such as *Peniophorella pallida* and *Lycoperdon perlatum*).

A total of 97 OTUs were classified as wood saprobes, which would include all of the typical wood decay fungi. Table 8 shows a breakdown of those fungi classified as wood saprobes by Funguild and their decay traits (soft, brown or white) with their occurrence across treatments.

A total of 14 brown rots, 38 soft rots and 37 white rots were identified and characterized from the soil samples (an additional 8 OTUs, containing 4 species of *Psathyrella, Crepidotus* sp. and *Simocybe* spp., were not characterized according to decay traits). Overall, a relatively low number of known wood decay fungi were obtained from our sampling, but these results will serve as important baseline data as we begin to focus on fungi that colonize and persist in solid wood and also highlights the importance of saprophytic fungi in the decay process. At both sites, higher abundance of wood rot fungi was noted for the exposed areas compared to unexposed areas. A relatively small number of wood saprobes were recovered in our sampling. Many of these species are well studied wood rot fungi or at least the genera, except for *Trechispora* spp. which is more common on decaying wood debris on the forest floor than solid wood. The less recognized species could be more difficult to culture or less likely to show fruiting in the field or even occur in later stages of decay. Our results could lead to more studies on these lesser emphasized wood rot fungi, especially in the preservative tolerance field. Proximity to remaining field stakes in the plot was not taken into consideration in this study, but could be a significant source of variation in these types of studies. Future studies to address these effects would provide much needed information on whether or not nutrient availability (i.e., untreated wood stakes) stimulates below ground fungal activity. Future studies will include more detailed information on wood preservative residues and breakdown products in the soil and would be an obvious next step in correlating long term preservative breakdown with fungal community composition. Another important consideration is whether present soil fungi directly correlate to wood decay. Continued studies will investigate transfer of soil fungi into untreated pine stakes and eventually preservative treated pine in field exposure.

#### Additional copper tolerant fungi

A secondary objective of this study was to screen for the presence of copper tolerant basidiomycete fungi in copper exposed sites. Several fungal genera and species were identified with published accounts of copper tolerance. These species were not abundant enough to be classified as indicator species but still exhibited patterns of presence consistent with prior literature. The abundance and distribution of these fungal OTUs by can be found in Table 9.

**Table 9:** Breakdown of Basidiomycete fungi classified as wood saprobes by Funguild highlighting the effects due to treatment on wood decay fungal diversity.

**Species**	**MS**			**WI**			
	**Mixed**	**Copper**	**Control**	**Mixed**	**Copper**	**Control**	**Decay mode**
*Trechispora* sp.	0	0	0	3,783	301	1601	White Rot
*Trechispora invisitata*	0	3,131	0	0	0	0	White Rot
*Trechispora* sp.	0	0	0	2,927	0	0	White Rot
*Trechispora* sp.	0	0	0	1,292	11	0	White Rot
*Ganoderma applanatum*	0	0	0	111	223	446	White Rot
*Ceriporiopsis* sp.	264	0	0	0	0	0	White Rot
*Heterobasidion* sp.	92	16	3	0	0	0	White Rot
*Trechispora* sp.	0	0	0	56	38	0	White Rot
*Trechispora* sp.	0	0	0	40	4	0	White Rot
*Ganoderma lucidum*	9	18	10	1	0	0	White Rot
*Bjerkandera* sp.	0	37	0	0	0	0	White Rot
*Sistotrema* sp.	25	1	3	0	0	4	White Rot
*Trechispora* sp.	1	0	28	0	0	0	White Rot
*Gymnopilus liquiritiae*	0	10	0	0	0	0	White Rot
*Bjerkandera adusta*	0	0	0	1	0	5	White Rot
*Trechispora* sp.	5	0	0	0	0	0	White Rot
*Irpex* sp.	0	1	2	0	0	1	White Rot
*Trechispora* sp.	1	1	2	0	0	0	White Rot
*Perenniporia* sp.	0	0	0	0	0	3	White Rot
*Ganoderma* sp.	0	0	0	0	1	2	White Rot
*Perenniporia subacida*	1	1	1	0	0	0	White Rot
*Phlebia* sp.	0	3	0	0	0	0	White Rot
*Trechispora* sp.	1	1	1	0	0	0	White Rot
*Ischnoderma resinosum*	0	0	0	0	0	2	White Rot
*Rigidoporus* sp.	0	1	1	0	0	0	White Rot
*Trechispora* sp.	0	0	0	2	0	0	White Rot
*Schizophyllum* sp.	0	0	2	0	0	0	White Rot
*Trechispora* sp.	0	0	1	0	0	0	White Rot
*Ganoderma lucidum*	0	0	0	0	1	0	White Rot
*Sistotrema* sp.	0	1	0	0	0	0	White Rot
*Daedaleopsis confragosa*	0	0	0	0	0	1	White Rot
*Polyporus squamosus*	0	0	0	1	0	0	White Rot
*Phlebia radiata*	0	0	0	0	0	1	White Rot
*Trichaptum* sp.	0	1	0	0	0	0	White Rot
*Bjerkandera* sp.	0	1	0	0	0	0	White Rot
*Trechispora* sp.	1	0	0	0	0	0	White Rot
*Ganoderma* sp.	0	1	0	0	0	0	White Rot
*Scytalidium lignicola*	0	1,610	0	0	0	0	Soft Rot
*Cladosporium herbarum*	0	0	0	397	102	998	Soft Rot
*Humicola* sp.	0	0	0	25	0	1,086	Soft Rot
*Trichoderma* sp.	71	179	106	277	17	13	Soft Rot
*Trichoderma* sp.	360	87	75	31	7	0	Soft Rot
*Scytalidium* sp.	26	45	197	0	0	0	Soft Rot
*Scytalidium* sp.	74	79	0	3	0	0	Soft Rot
*Scytalidium* sp.	34	75	0	0	0	0	Soft Rot
*Trichoderma crassum*	20	47	26	0	1	0	Soft Rot
*Phoma* sp.	0	0	0	27	1	21	Soft Rot
*Chalara* sp.	0	0	41	0	0	0	Soft Rot
*Stilbella fimetaria*	0	0	0	27	0	0	Soft Rot
*Alternaria infectoria*	0	0	0	3	5	16	Soft Rot
*Xylaria* sp.	11	1	0	0	0	0	Soft Rot
*Chalara* sp.	0	11	0	0	0	0	Soft Rot
*Scytalidium* sp.	10	0	0	0	0	0	Soft Rot
*Xylaria* sp.	0	2	8	0	0	0	Soft Rot
*Scytalidium* sp.	8	1	0	0	0	0	Soft Rot
*Trichoderma* sp.	0	0	0	7	0	0	Soft Rot
*Scytalidium* sp.	0	7	0	0	0	0	Soft Rot
*Coniothyrium* sp.	0	0	0	1	1	5	Soft Rot
*Trichoderma hamatum*	2	0	4	0	0	0	Soft Rot
*Trichoderma* sp.	1	1	2	0	0	1	Soft Rot
*Coniothyrium* sp.	0	0	0	0	0	3	Soft Rot
*Scytalidium* sp.	0	0	3	0	0	0	Soft Rot
*Scytalidium* sp.	0	0	0	3	0	0	Soft Rot
*Coniothyrium* sp.	0	0	0	0	2	0	Soft Rot
*Xylaria* sp.	2	0	0	0	0	0	Soft Rot
*Phoma* sp.	0	0	0	0	0	2	Soft Rot
*Cytospora diatrypelloidea*	2	0	0	0	0	0	Soft Rot
*Xylaria* sp.	0	0	0	0	2	0	Soft Rot
*Pyrenochaeta* sp.	0	1	0	0	0	0	Soft Rot
*Cytospora* sp.	0	1	0	0	0	0	Soft Rot
*Trichoderma* sp.	0	0	1	0	0	0	Soft Rot
*Chalara* sp.	0	0	0	0	1	0	Soft Rot
*Xylaria cornu-damae*	0	0	0	1	0	0	Soft Rot
*Chaetomium aureum*	0	1	0	0	0	0	Soft Rot
*Trichoderma* sp.	0	0	0	1	0	0	Soft Rot
*Psathyrella lyckebodensis*	0	0	0	18	13	0	NULL
*Psathyrella* sp.	0	0	0	6	3	1	NULL
*Crepidotus* sp.	1	1	0	0	0	1	NULL
*Psathyrella romellii*	0	0	0	0	2	0	NULL
*Psathyrella candolleana*	0	1	0	1	0	0	NULL
*Crepidotus* sp.	0	0	0	0	0	1	NULL
*Simocybe* sp.	1	0	0	0	0	0	NULL
*Psathyrella tenuicula*	1	0	0	0	0	0	NULL
*Postia guttulata*	0	0	0	957	22	0	Brown Rot
*Leucogyrophana olivascens*	0	589	0	0	0	0	Brown Rot
*Serpula himantioides*	3	69	0	0	0	0	Brown Rot
*Dacryobolus sudans*	9	0	0	0	0	0	Brown Rot
*Coniophora olivacea*	0	5	0	0	0	0	Brown Rot
*Sarcoporia* sp.	0	0	0	3	0	1	Brown Rot
*Ditiola radicata*	0	3	0	0	0	0	Brown Rot
*Coniophora* sp.	3	0	0	0	0	0	Brown Rot
*Coniophora* sp.	0	2	0	0	0	0	Brown Rot
*Gloeophyllum trabeum*	0	0	0	0	0	2	Brown Rot
*Tapinella panuoides*	0	2	0	0	0	0	Brown Rot
*Antrodia carbonica*	0	0	0	1	0	0	Brown Rot
*Fibroporia radiculosa*	1	0	0	0	0	0	Brown Rot
*Oxyporus latemarginatus*	0	0	0	0	0	1	Brown Rot

*At both sites, higher abundance of wood decay fungi was noted for the treated areas compared to untreated forested areas. Note that counts in the cells are total reads, not number of OTUs. A relatively small number of small wood saprobes were recovered in our sampling*.

The brown rot are most typically associated with copper tolerance and several taxa were found in copper exposed soils belonging to that group. *Postia guttulata* was only detected in preservative exposed soils in WI along with *Antrodia carbonica*. *Antrodia carbonica* has previously been documented as copper tolerant and *P. guttulata* is in the same genus as *Postia placenta*, which can also exhibit copper tolerance (De Groot and Woodward, 1999). The lack of brown rot detected in untreated areas in MS cannot be explained, however fungi characterized as white rot and soft rot fungi were detected. There were several brown rots only found in preservative treated areas in MS. *Fibroporia radiculosa* is a very well-known cooper tolerant brown rot fungus (Clausen and Jenkins, 2011) and was detected in the mixed preservative post plots in MS. This fungus is also common in our copper treated samples exposed to soil in MS, but wasn't as prevalent as expected in these soil samples. *Dacryobolus sudans* and *Coniophora* sp. were also detected in the mixed preservative plots. *Coniophora puteana* is a common preservative tolerant fungus, especially on creosote treated members and *Dacryobolus sudans* has been shown to at least colonize PAH contaminated soils (Tornberg et al., 2003) also typically associated with creosote contaminated soils. *Leucogryaphana olivasceans* was also abundant in copper plots in MS, but no prior literature describing copper tolerance of this species was found. *Serpula himantoides* was detected in both mixed preservative and copper exposed plots in MS and is closely related to *S. lacrymans*, which has published records of copper tolerance (Watkinson and Eastwood, 2012).

There were several unique white rot fungal OTUs only present in the copper exposed areas in MS: *Trechispora*, both sp. and *T. invisitata, Heterobasidion* sp., *Ganoderma lucidum, Gymnopilus liquirtiae, Phlebia* sp., *Sistotrema* sp., and *Trichaptum* sp. Several OTUs identified as separate OTUs of *Trechispora* sp. were also found in WI, but were not limited to copper or mixed preservative exposed soils. *Ceriporiopsis* sp. was detected only in mixed preservative exposed soils in MS. *C. subvermispora* has been screened its ability to break down recalcitrant compounds in soil and liquid culture such as poly chlorinated biphenyls (PCBs) (Valentín et al., 2013), azoles (Woo et al., 2010) and pentachlorophenol (Machado et al., 2005) and is one of four fungi patented for use in mycoremediation of preservative treated wood waste (Lamar et al., 1995).

Most of the wood saprobes classified in the funguild analysis were classified as soft rot fungi. Many of these were cosmopolitan in their distribution among the different treatment exposures (ex. *Trichoderma* sp., *Scytalidium* sp., and *Humicola* sp.). Notable unique occurrences of soft rot fungi include: *Scytalidium lignicola* (detected only in MS copper treated), *Stilbella fimetaria* (detected only in WI mixed treatments). *Chaetomium aureum* was detected solely in copper treatments in MS and members of this genus have been used to pre-inoculate preservative treated wood blocks to initiate preservative breakdown (Duncan and Deverall, 1964).

## Conclusion

Amplicon-based sequence analysis provides a valuable tool for detailed characterization of soil fungal communities. The results of this study suggest that long-term wood preservative exposure can impact soil community species composition and alter functional guild composition. Both site and treatment had significant effects on soil fungal community composition. Chemical analysis indicated little to no difference in metal concentrations compared to untreated soils, suggesting either leaching or complete breakdown of any remaining preservative residues in these studies. Exposure to the mixed treatment stimulated an increase in terrestrial ectomycorrhizal fungi, suggesting this guild of fungi may be more adapted to breakdown of residual wood preservatives than the wood decay fungi frequently used for bioremediation explorations. Wood decay fungi were often very specific to one site or the other, suggesting that local decay biota are present at a site, which may challenge the notion of generalized laboratory decay tests to determine efficacy of wood protectants.

## Author contributions

GK was responsible for design of the experiments, data gathering and analysis, and preparation of the manuscript. WH assisted in the planning stages of the experiments, offered technical expertise for metagenomic analyses and provided input in the preparation of the manuscript. AB assisted in the planning stages of the experiments, processed soil samples for analysis and assisted in preparation of the manuscript. JP provided technical expertise on amplicon sequencing data analysis and assisted in preparation of the manuscript. MJ provided ecological statistics of sequencing data and assisted in preparation of the manuscript. DL provided assistance on design of the experiment and manuscript preparation.

### Conflict of interest statement

The authors declare that the research was conducted in the absence of any commercial or financial relationships that could be construed as a potential conflict of interest.

## Supplementary material

Supplementary Figure S1 Taxonomic diversity of samples at the Phylum level.

Supplementary Figure S2 Taxonomic diversity of samples at the Class level.

Supplementary Figure S3 Taxonomic diversity of samples at the Order level.

Supplementary Figure S4 Taxonomic diversity of samples at the Family level.

Supplementary Figure S5 Taxonomic diversity of samples at the Genus level.
