# Supplementary figures and images for: Amplicon-Based Sequencing of Soil Fungi from Wood Preservative Test Sites
Source: Front Microbiol. 2017 Oct 18;8:1997. doi: 10.3389/fmicb.2017.01997 (PMC5651271; doi:10.3389/fmicb.2017.01997)

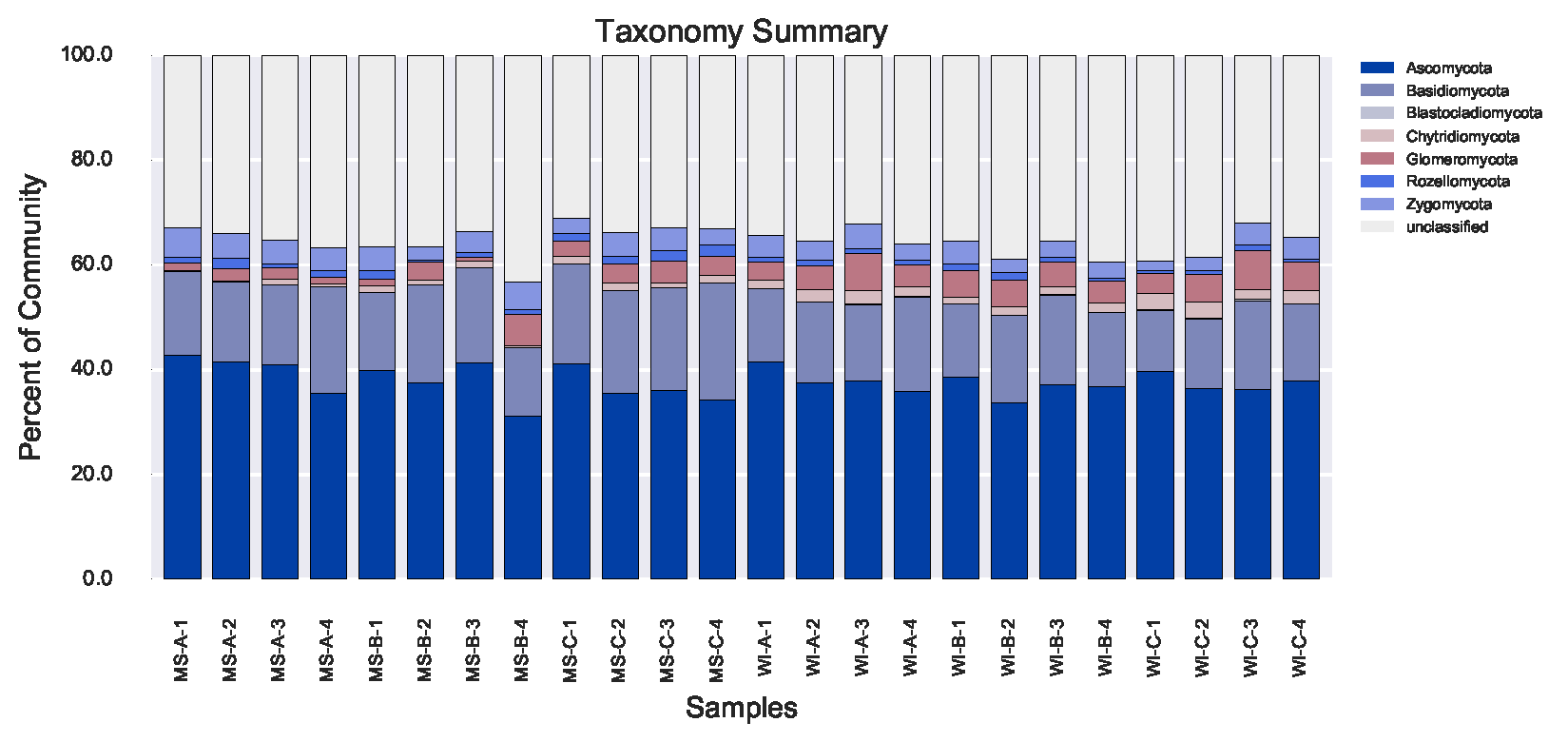

Supplement: Supplementary Figure S1 — Taxonomic diversity of samples at the Phylum level. [file Image1.TIFF]

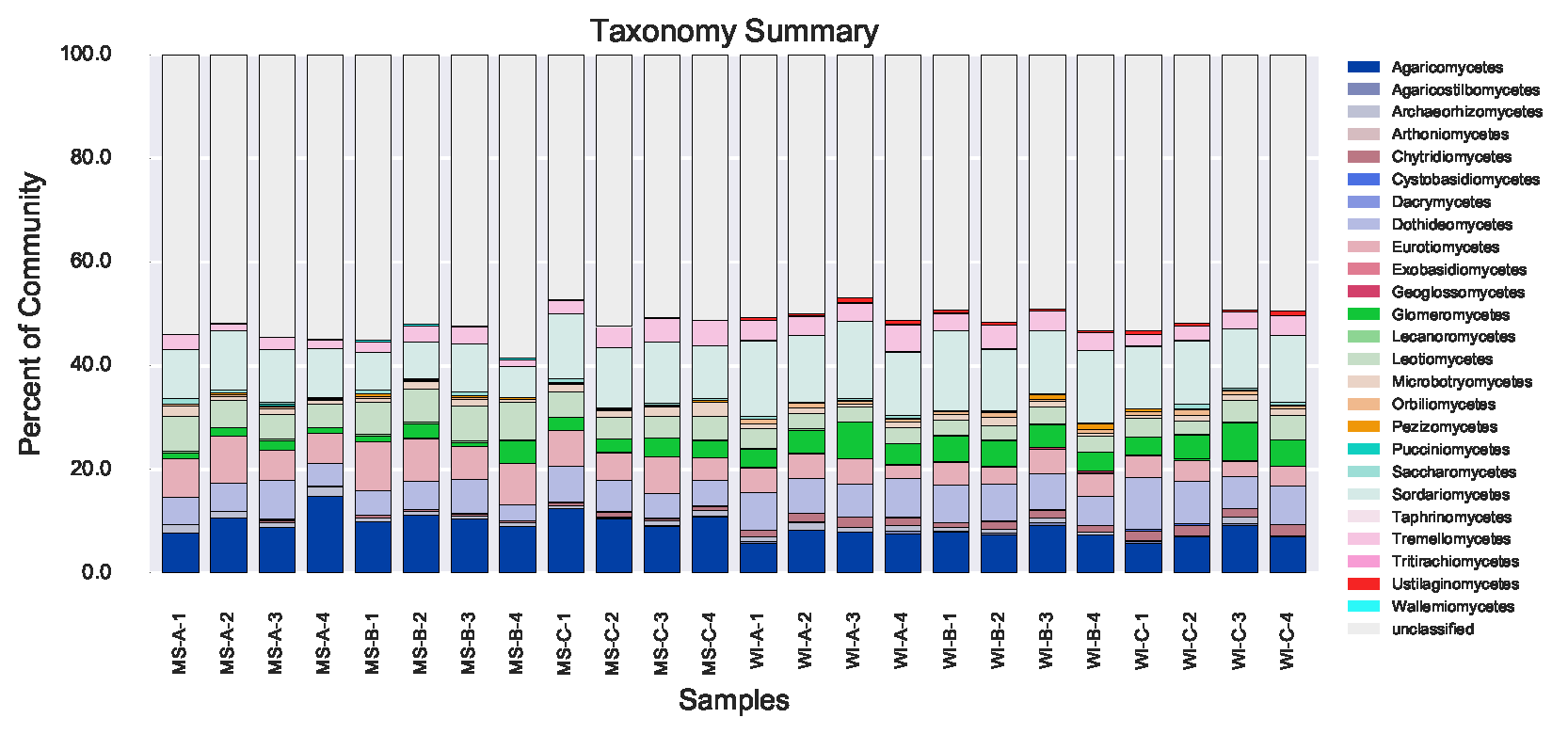

Supplement: Supplementary Figure S2 — Taxonomic diversity of samples at the Class level. [file Image2.TIF]

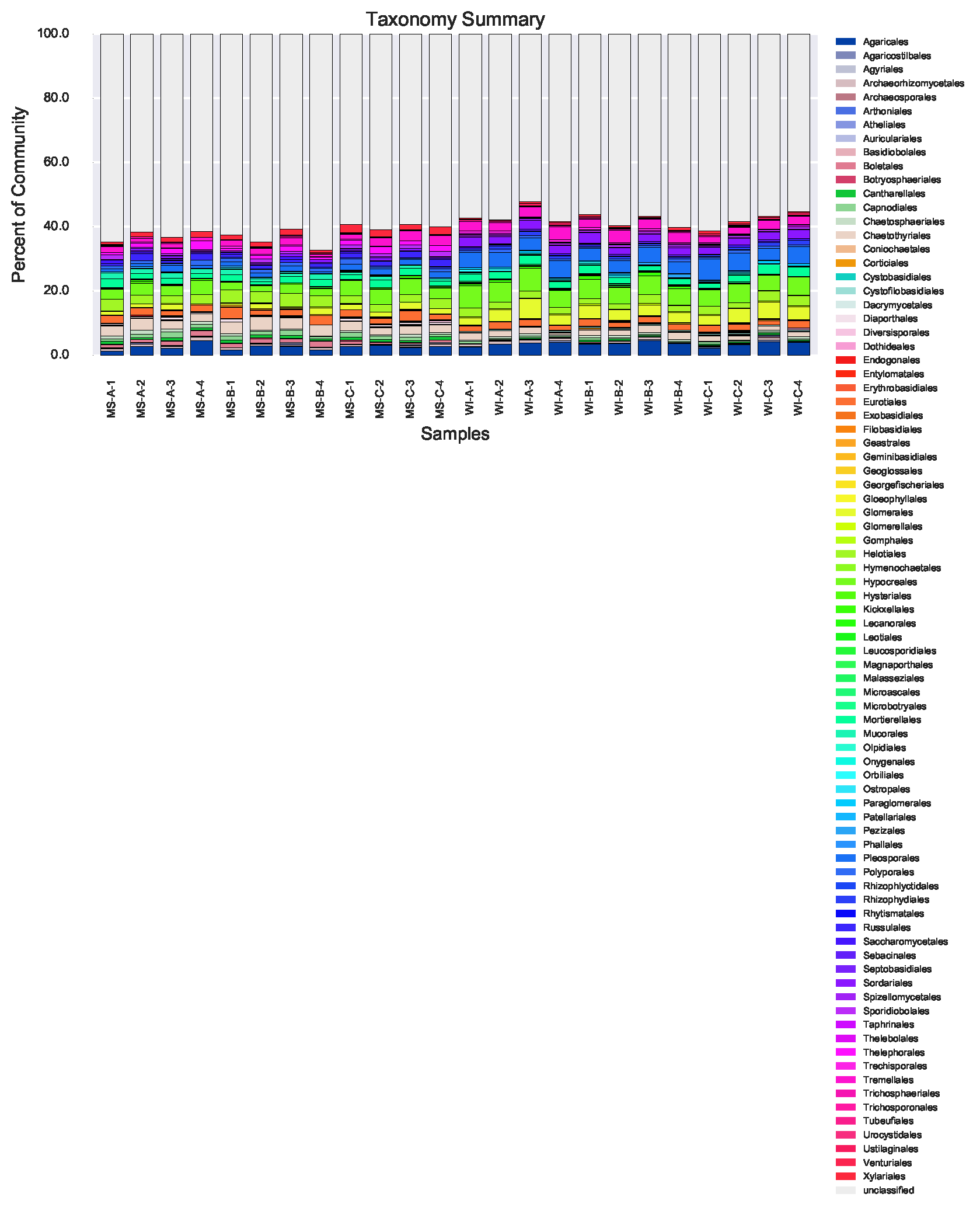

Supplement: Supplementary Figure S3 — Taxonomic diversity of samples at the Order level. [file Image3.TIF]

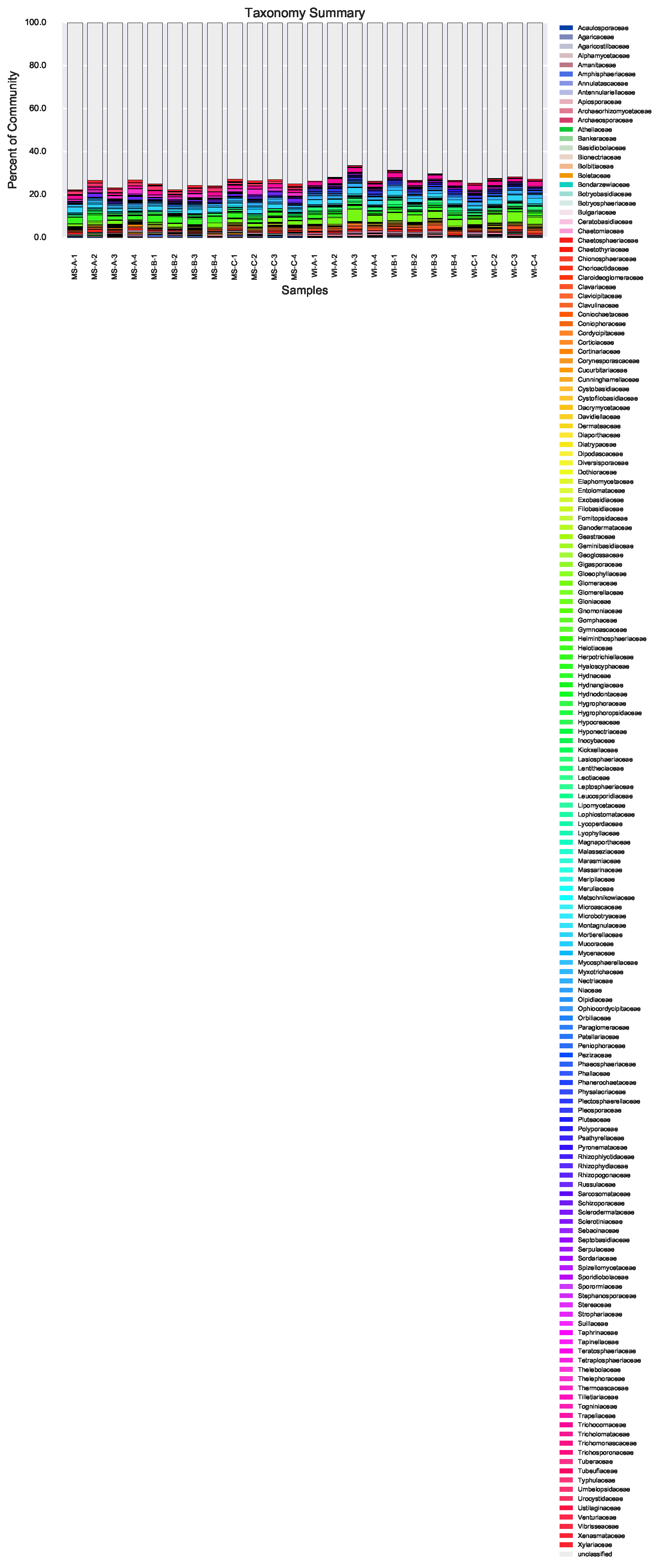

Supplement: Supplementary Figure S4 — Taxonomic diversity of samples at the Family level. [file Image4.TIF]

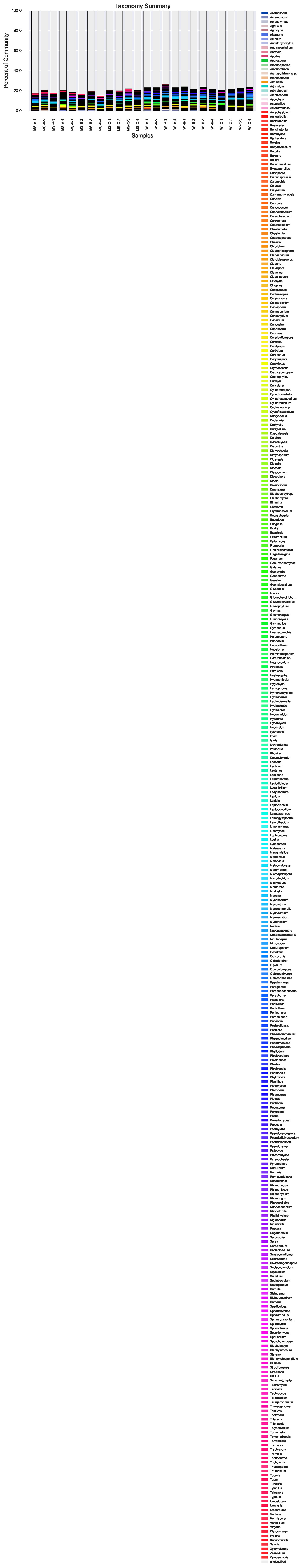

Supplement: Supplementary Figure S5 — Taxonomic diversity of samples at the Genus level. [file Image5.TIF]
